# Does MRI add value in selecting patients for thrombectomy beyond the 6 h window? A matched-control analysis

**DOI:** 10.3389/fneur.2023.1135624

**Published:** 2023-04-17

**Authors:** Huiran Cheng, Zequan Yu, Gaoting Ma, Anxin Wang, Baixue Jia, Xu Tong, Ning Ma, Feng Gao, Dapeng Mo, Ligang Song, Sun Xuan, Xiaochuan Huo, Zi-Xian Zhang, Zeguang Ren, Zhongrong Miao

**Affiliations:** ^1^Department of Neurosurgery, The People's Hospital of Anyang City, Anyang, China; ^2^Department of Interventional Neuroradiology, Beijing Tiantan Hospital, Capital Medical University, Beijing, China; ^3^Department of Neurology, Xuanwu Hospital, Capital Medical University, Beijing, China; ^4^China National Clinical Research Center for Neurological Diseases, National Clinical Research Center for Neurological Diseases, Beijing, China

**Keywords:** thrombectomy, stroke, LVO = large vessel occlusion, computed tomography, magnetic resonance imaging

## Abstract

**Background:**

Controversy exists regarding the need of advanced imaging for patient selection in the extended window.

**Aims:**

To analyze the effect of initial imaging modalities on clinical outcomes of patients underwent MT in the extended window.

**Methods:**

This was a retrospective analysis of a prospective registry, the Endovascular Treatment Key Technique and Emergency Workflow Improvement of Acute Ischemic Stroke (ANGEL-ACT) registry which was conducted at 111 hospitals between November 2017 and March 2019 in China. Primary study cohort and Guideline like cohort were identified, in each cohort, two imaging modalities for patient selection in 6 to 24 h window were defined: (1) NCCT ± CTA, (2) MRI. Guideline-like cohort were further screened based on key features of the DAWN and DEFUSE 3 trials. The primary outcome was 90 day mRS. The safety outcomes were sICH, any ICH and 90-day mortality.

**Results:**

After adjusting for covariates, there were no significant differences in 90 day mRS or any safety outcomes between two imaging modalities groups in both cohorts. All outcome measures of mixed-effects logistic regression model were consistent with propensity score matching model.

**Conclusion:**

Our results indicate that patients presented with anterior large vessel occlusion in the extended time window can potentially benefit from MT even in the absence of MRI selection. This conclusion needs to be verified by the prospective randomized clinical trials.

## Introduction

The efficacy of endovascular treatment for acute large vessel occlusion (LVO) strokes presenting within the first 6 h after symptom onset has been demonstrated in 7 randomized clinical trials (1–7). In addition, the DAWN (DWI or CTP Assessment With Clinical Mismatch in the Triage of Wake-Up and Late Presenting Strokes Undergoing Neurointervention With Trevo) and DEFUSE 3 (Endovascular Therapy Following Imaging Evaluation for Ischemic Stroke) trials demonstrated robust benefit of mechanical thrombectomy (MT) in the 6 to 24 h window over medical management alone, but with certain imaging criteria of ischemic core (8, 9). Although the stringent imaging criteria of advanced imaging in the extended window are recommended by current guidelines, mandated criteria of ischemic core and penumbra on computed tomography perfusion (CTP) or brain magnetic resonance imaging (MRI) have been criticized for being overly selective and may lead to under-treated (10–13). Recent studies have shown that CTP acquisition did not improve outcomes in patients treated in the extended window (14), and CTP may not be reliable to exclude patients who will not benefit from intra-arterial therapy (10). However, paucity of prospective or retrospective data compared the outcomes of MT in patients selected by non-contrast computed tomography (NCCT) ± CT angiography (CTA) versus those selected by MRI in the extended window. In this study, we sought to compare the effect of NCCT ± CTA with MRI imaging modality on clinical outcomes of MT in patients who presented 6–24 h after symptom onset in a large, prospective, and national endovascular stroke thrombectomy registry in China (15).

## Hypothesis

There is no significant difference in terms of primary outcomes and safety outcomes across the MRI and NCCT ± CTA groups.

## Methods

### Study population

This study is a retrospective sub-analysis of a prospective multicenter registry study, the Endovascular Treatment Key Technique and Emergency Workflow Improvement of Acute Ischemic Stroke (ANGEL-ACT) Registry (Registration-RUL: https://www.clinicaltrials.gov; Unique identifier: NCT03370939). The ANGEL-ACT registry enrolled consecutive patients with acute ischemic stroke attributed to the large-vessel occlusion underwent endovascular therapy within 24 h after symptom onset (or last known well [LKW]) in 111 hospitals of China. Detailed inclusion and exclusion criteria have been reported previously (15). Written informed consent was obtained from all patients or their legally authorized representatives. The original study protocol was approved by a central medical ethics committee and the research board of each participating center.

The Primary study cohort was comprised of all ANGEL-ACT patients presented in the extended time window, with acute large vessel occlusion involving the intracranial carotid artery (ICA), or either the M1 or M2 segments of the middle cerebral artery, premorbid modified Rankin Score (mRS) of 0 to 2, and LKW to arterial puncture time of 6 to 24 h. A homogenous subgroup of these patients was defined as the “Guideline-like cohort” based on the key clinical and demographic features of the DAWN or DEFUSE 3 trials (age ≥ 18 years, baseline National Institutes of Health Stroke [NIHSS] score ≥ 10, ICA or M1 occlusion, and premorbid mRS score 0–1). Patients with missing baseline mRS, NIHSS, core infarct volume in the MRI group and occlusion sites other than ICA, M1 or M2 segments of the middle cerebral artery were excluded from this analysis.

The study cohorts were categorized according to the pretreatment imaging modalities: (1) NCCT ± CTA. (2) brain MRI (T1 + T2 + fluid-attenuated inversion recovery [FLAIR] + diffusion-weighted imaging [DWI] ± magnetic resonance angiography [MRA]). Patients selected by CTP in the ANGEL-ACT registry were excluded, because the small sample size (n = 44) may exclude a large number of patients in the other cohorts after PS matching, which may cause tremendous selection bias.

### Imaging analysis and outcomes

All images were independently assessed by core lab staff blinded to clinical and outcome data. Imaging assessment included early ischemic changes on NCCT or DWI using Alberta Stroke Program Early CT Score (ASPECTS) for anterior circulation strokes (ACSs), location of occlusion site, baseline and post-procedural score on the modified Thrombolysis in Cerebral Infarction (mTICI) scale, intracranial hemorrhage (ICH), and symptomatic ICH (sICH). In the MRI group, the ischemic core volume was defined as lesions on DWI or an apparent diffusion coefficient [ADC] threshold of <620 × 10^−3^ mm/s (16). The assessments were carried out using a fully automated image processing software (RAPID, iSchemaView, Menlo Park, CA, United States) after limited core laboratory reader quality check and standardization of image parameters.

The primary outcome is measured with an ordinal score of mRS at 90 days (shift analysis), mRS scores were evaluated using a standardized telephone interview performed by trained investigators blinded to the baseline and procedural data (17). The secondary outcomes include the proportions of mRS 0 to 1, 0 to 2, and 0 to 3 at 90 days, and dramatic clinical improvement (DCI) which is defined as NIHSS score ≤ 1 at 24 h or ≥ 10 points decrease within 24 h (18). Safety outcomes include any ICH, sICH (according to the Heidelberg Bleeding Classification) (19), and mortality at 90 days.

### Statistical analysis

Patients’ baseline characteristics and outcome variables were analyzed and presented using percentages, median, and interquartile ranges. Statistical significance for intergroup differences was assessed by the Fisher exact test for categorical variables and by Kruskal-Wallis test for continuous variables. Propensity-matched analysis was performed in order to improve comparability between the two groups. Pre-exposure baseline characters in Table 1 (which include age, gender, baseline mRS, baseline NIHSS, NCCT/DWI ASPECTS score and onset-puncture time) were used to generate the propensity scores. The patients were allocated using a 1:1 matching protocol without replacement (greedy-matching algorithm), with a caliper width ≤ 0.1 of the SD of the logit of the propensity scores. For comparing outcome measures between the cohorts in the prematched and postmatched population, the odds ratios (aOR), along with their 95% confidence intervals (CIs), were calculated using binary or ordinal logistic regression model, and multivariable models were used to adjust for potential confounders. The cofounders include age, ASPECTS, last known well to arterial puncture time, occlusion site, successful reperfusion and centers. All outcome measures between the two groups in the total population using mixed-effects logistic regression models adjusted for the variables with a significant difference of *p* < 0.05 and center as random effect. Patients with missing data were excluded from further analysis. All analyses were performed using SAS version 9.4 software (SAS Institute, Cary, NC, United States). A probability value of <0.05 was regarded as significant.

**Table 1:** Baseline and procedural characteristic of the extended time window patients according to imaging selection modality.

Primary study cohort	Before PS matching				After PS matching			
	NCCT±CTA (*n* = 196)	MRI (*n* = 228)	Standardized difference (%)	*p* value	NCCT±CTA (*n* = 102)	MRI (*n* = 102)	Standardized difference (%)	*p* value
Age, median (IQR), years	65 (54–72)	63 (54–70)		0.236	66 (54–73)	63 (55–70)	−2.8	0.460
Male sex	133 (67.9)	158 (69.3)		0.754	69 (67.6)	70 (68.6)	−2.1	1.000
Baseline mRS score				0.760			−9.3	0.659
0	173 (88.3)	198 (86.8)			89 (87.3)	92 (90.2)		
1	22 (11.2)	27 (11.8)			13 (12.8)	10 (9.8)		
2	1 (0.5)	3 (1.3)			0 (0.0)	0 (0.0)		
Baseline NIHSS score, median (IQR)	16 (11–20)	14 (10–18)		0.004	14 (12–19)	15 (11–18)	−3.1	0.770
NCCT/DWI ASPECTS, median (IQR)	10 (8–10)	7 (6–8)		<0.001	8 (6–10)	8 (7–9)	−1.6	0.629
Volume, median (IQR), ml	–	17 (9–33)	–	–	–	12 (6–19)		–
Occlusion site				0.835			10.4	0.769
Internal carotid artery	60 (30.6)	63 (27.6)			31 (30.4)	27 (26.5)		
MCA-M1	117 (59.7)	147 (64.5)			61 (59.8)	66 (64.7)		
MCA-M2	19 (9.7)	18 (7.9)			10 (9.8)	9 (8.8)		
Intravenous thrombolysis	47 (24.0)	51 (22.4)		0.730	21 (20.6)	23 (22.6)	4.8	0.865
General anesthesia	126 (64.3)	129 (56.6)		0.112	66 (64.7)	59 (57.8)	14.1	0.389
Successful reperfusion^a^	173 (88.3)	200 (87.7)		0.882	90 (88.2)	95 (93.1)	16.9	0.336
Pass number of thrombectomy, median (IQR)	2 (1–3)	1 (1–3)		0.832	1 (1–2)	1 (1–2)	8.8	0.342
LKW-to-arterial puncture time, median (IQR), min	480 (401–616)	518 (420–747)		0.015	540 (425–698)	485 (410–734)	−4.0	0.388
Puncture-to-reperfusion time, median (IQR), min	88 (55–130)	86 (52–130)		0.708	88 (62–123)	75 (52–120)	−2.2	0.207
Guideline-like cohort	Before PS matching				After PS matching			
	NCCT±CTA (*n* = 157)	MRI (*n* = 146)	Standardized difference (%)	*p* value	NCCT±CTA (*n* = 73)	MRI (*n* = 73)	Standardized difference (%)	*p* value
Age, median (IQR), years	65 (53–71)	63 (51–70)		0.251	65 (54–73)	63 (51–69)	−13.1	0.295
Male sex	106 (67.5)	100 (68.5)		0.902	47 (64.4)	54 (74.0)	−20.9	0.282
Baseline mRS score				0.439			−8.3	0.802
0	142 (90.5)	128 (87.7)			63 (86.3)	65 (89.0)		
1	15 (9.6)	18 (12.3)			10 (13.7)	8 (11.0)		
Baseline NIHSS score, median (IQR)	16 (12–21)	14 (11–18)		0.004	15 (12–19)	15 (12–19)	−7.0	0.948
NCCT/DWI ASPECTS, median (IQR)	10 (8–10)	7 (7–9)		<0.001	8 (7–10)	8 (7–9)	0.0	0.975
Volume, median (IQR), ml	–	16 (9–32)		–	–	13 (6–24)		–
Occlusion site				0.406			30.2	0.104
Internal carotid artery	51 (32.5)	41 (28.1)			27 (37.0)	17 (23.3)		
MCA-M1	106 (67.5)	105 (71.9)			46 (63.0)	56 (76.7)		
Intravenous thrombolysis	35 (22.3)	32 (21.9)		1.000	14 (19.2)	13 (17.8)	−3.5	1.000
General anesthesia	99 (63.1)	87 (59.6)		0.557	46 (63.0)	47 (64.4)	−2.9	1.000
Successful reperfusion	139 (88.5)	130 (89.0)		1.000	67 (91.8)	63 (86.3)	−17.6	0.428
Pass number of thrombectomy, median (IQR)	2 (1–3)	1 (1–2)		0.385	1 (1–2)	2 (1–3)	13.4	0.329
LKW-to-arterial puncture time, median (IQR), min	470 (400–600)	521 (420–747)		0.007	498 (415–665)	500 (420–715)	10.9	0.719
Puncture-to-reperfusion time, median (IQR), min	88 (54–130)	79 (53–123)		0.692	90 (54–10)	77 (53–129)	0.9	0.796

ASPECTS, Alberta stroke program early computed tomography score; CTA, computed tomography angiography; DWI, diffusion-weighted imaging; IQR, interquartile range; LKW, last known well; M1, M1 segment; M2, M2 segment; MCA, middle cerebral artery; MRI, magnetic resonance imaging; mRS, modified Rankin scale; NCCT, non-contrast computed tomography; NIHSS, national institutes of health stroke scale; PS, propensity score.

a Successful reperfusion was defined as a modified Thrombolysis in Cerebral Infarction score of 2b or 3.

## Results

**Figure 1:**
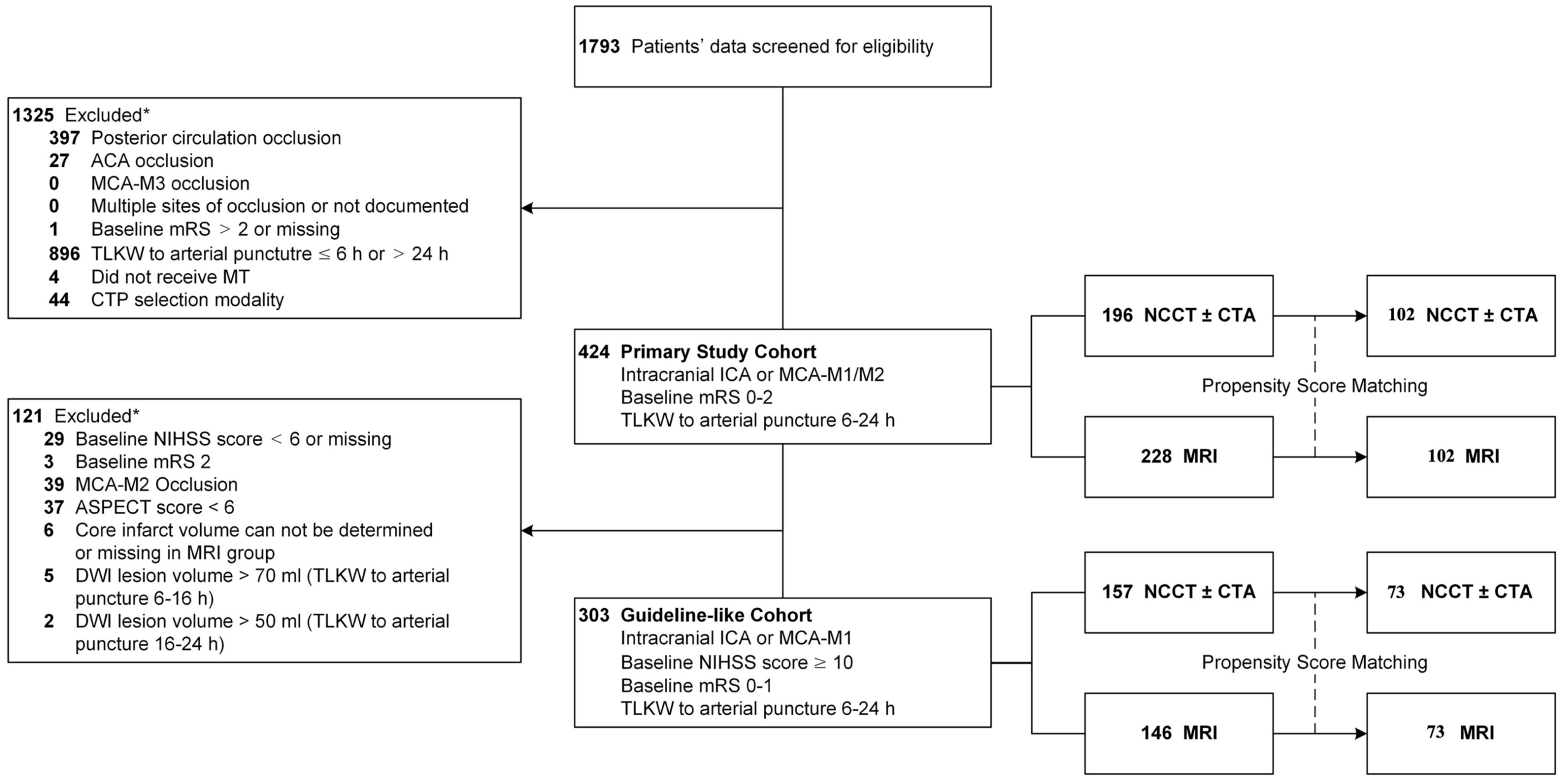
Flow diagram showing study population screening, eligibility, and inclusion.

Of the 1793 patients enrolled in the ANGEL-ACT registry, 468 patients (26.1%) underwent MT in the 6 to 24 h window. In the primary study cohort, 1 patient was excluded due to missing baseline mRS, while 29 patients were excluded due to missing baseline NIHSS score in the guideline-like cohort. 424 patients (23.6%) were included in the Primary study cohort and a total of 303 patients (16.9%) were included in the Guideline like cohort. In the Primary study cohort, 196 (46.2%) patients underwent NCCT ± CTA alone and 228 (67.9%) patients underwent MRI. After PS matching, 228 patients were matched, with 109 patients in each group. In the Guideline-like cohort, 157 patients (51.8%) underwent NCCT ± CTA alone, and 146 (48.2%) underwent MRI, After PS matching, 156 patients were matched, with 78 patients in each group. This process is shown in Figure 1. All patients enrolled in the participating centers were transferred to the emergency room for initial triage and no patients were directly transferred to the angiography suite.

### Baseline characteristics

Baseline characteristics are shown in Table 1. Before PS matching, in the primary study cohort, NCCT ± CTA group patients had higher baseline NIHSS scores (16 [11–20] versus 14 [10–18]; *p* = 0.004), higher ASPECTS (10 [8–10] versus 7 [6–8]; P <0.001), and shorter LKW to arterial puncture times (480 [401–616] versus 518 [420–747]; *p* = 0.015). Guideline-like cohort showed similar characteristics with NCCT ± CTA group had higher baseline NIHSS scores (16 [12–21] versus 14 [11–18]; p = 0.004), higher ASPECTS (10 [8–10] versus 7 [7–9]; P < 0.001), and shorter LKW to arterial puncture time (470 [400–600] versus 521 [420–747]; *p* = 0.007). All baseline characteristics are well-balanced after PS matching as is shown in Table 1.

### Outcome measures

**Figure 2:**
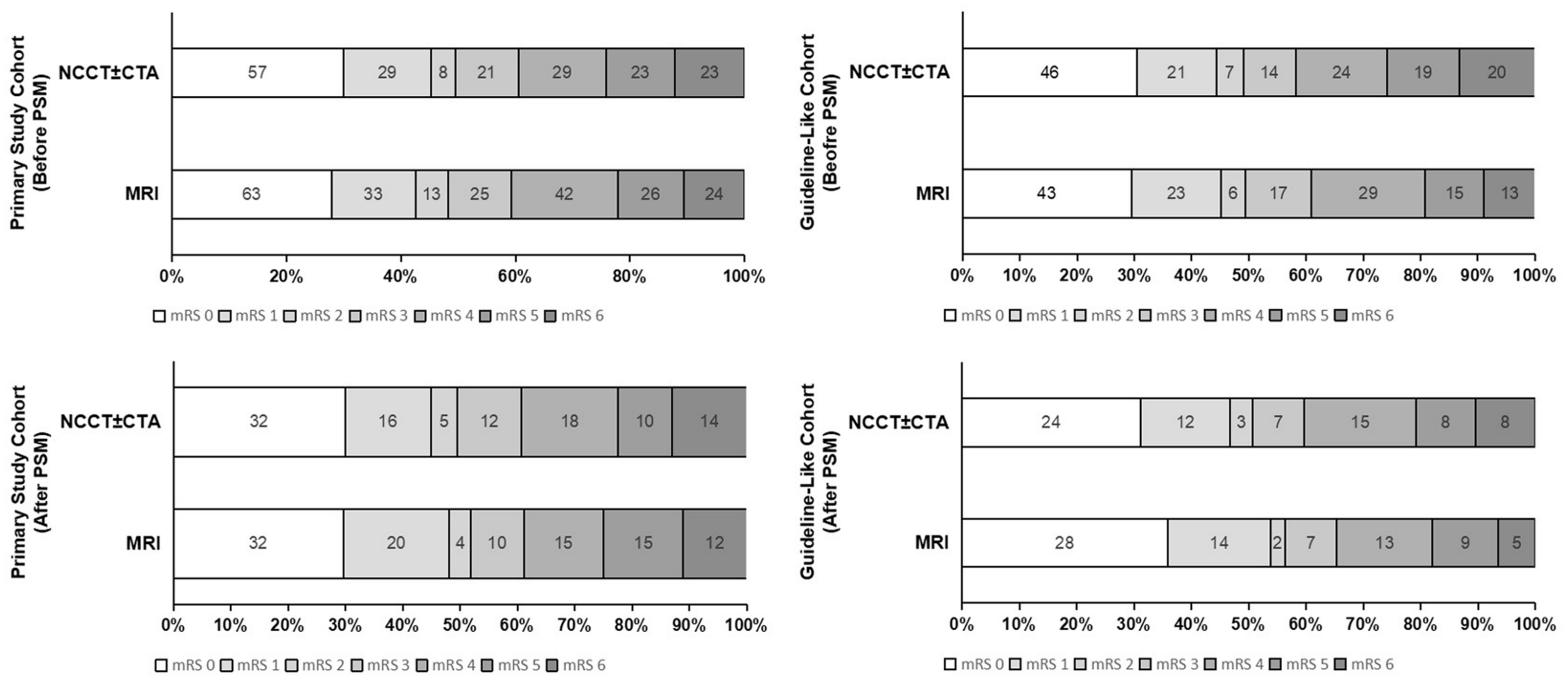
Modified Rankin Scale scores at 90 days before and after PSM.

A comparison of outcome measures is shown in Table 2. The shift on the 90-day mRS score is depicted in Figure 2. In the primary study cohort, after adjusting for covariates before PS matching, there were no significant difference in terms of 90-day functional disability (ordinal mRS shift: aOR 1.01, 95% CI [0.61–1.70], adjusted *p* value [a*P*] = 0.956), mRS 0-1(aOR 0.99, 95% CI [0.50–1.95], a*P* = 0.971), mRS 0–2 (aOR 0.98, 95% CI [0.52–1.88], a*P* = 0.974), mRS 0–3 (aOR 0.90, 95% CI [0.47–1.71], a*P* = 0.744), and DCI (aOR 1.22, 95% CI [0.50–2.99], a*P* = 0.666) across the MRI and the NCCT ± CTA groups. There were no significant difference in rates of ICH (27.2% vs. 23.0%, a*P* = 0.936), sICH (10.0% vs. 5.9%, a*P* = 0.889), and 90-day mortality (10.6% vs. 12.1%, a*P* = 0.724) across MRI versus NCCT ± CTA alone patients neither. The results of Guideline-like cohort were similar with that of the primary study cohort.

**Table 2:** Clinical and Radiographic Outcomes of the Cohorts.

Primary study cohort	Before PS matching						After PS matching			
	NCCT±CTA	MRI	Unadjusted analysis		Adjusted analysis^a^		NCCT±CTA	MRI	Unadjusted analysis	
	(*n* = 196)	(*n* = 228)	OR (95% CI)	*p* value	OR^a^ (95% CI)	*p* value	(*n* = 102)	(*n* = 102)	OR (95% CI)	*p* value
*Primary outcome*										
mRS at 90 d, median (IQR)	3 (0–4)	3 (0–4)	0.97 (0.69–1.36)	0.845	1.01 (0.61–1.70)	0.956	3 (0–4)	2 (0–4)	1.25 (0.76–2.04)	0.379
*Secondary outcomes*										
mRS 0–1 at 90 d	85/190 (45.3)	96/226 (42.5)	0.89 (0.61–1.32)	0.568	0.99 (0.50–1.95)	0.971	43/98 (43.9)xxx	46/101 (45.5)	1.07 (0.61–1.87)	0.813
mRS 0–2 at 90 d	94/190 (49.5)	109/226 (48.2)	0.95 (0.65–1.40)	0.801	0.98 (0.52–1.88)	0.974	46/98 (46.9)	53/101 (52.5)	1.25 (0.72–2.18)	0.436
mRS 0–3 at 90 d	115/190 (60.5)	134/226 (59.3)	0.95 (0.64–1.41)	0.798	0.90 (0.47–1.71)	0.744	59/98 (60.2)	67/101 (66.3)	1.30 (0.73–2.32)	0.370
DCI^b^	32/179 (17.9)	36/211 (17.1)	0.95 (0.56–1.60)	0.832	1.22 (0.50–2.99)	0.666	18/96 (18.8)	23/95 (24.2)	1.38 (0.69–2.77)	0.359
*Safety outcomes*										
Death within 90 d	23/190 (12.1)	24/226 (10.6)	0.86 (0.47–1.58)	0.634	0.89 (0.47–1.70)	0.724	13/98 (13.3)	8/101 (7.9)	0.56 (0.22–1.42)	0.224
Any ICH	43/187 (23.0)	60/221 (27.2)	1.25 (0.80–1.96)	0.336	0.97 (0.42–2.22)	0.936	21/97 (21.7)	25/99 (25.3)	1.22 (0.63–2.37)	0.552
Symptomatic ICH^c^	11/186 (5.9)	22/220 (10.0)	1.77 (0.83–3.75)	0.138	1.07 (0.42–2.69)	0.889	7/97 (7.2)	5/98 (5.1)	0.69 (0.21–2.26)	0.541
Guideline-like cohort	Before PS Matching						After PS Matching			
	NCCT±CTA	MRI	Unadjusted analysis		Adjusted analysis^a^		NCCT±CTA	MRI	Unadjusted analysis	
	(*n* = 157)	(*n* = 146)	OR (95% CI)	*p* value	OR^a^ (95% CI)	*p* value	(*n* = 73)	(*n* = 73)	OR (95% CI)	*p* value
*Primary outcome*										
mRS at 90 d, median (IQR)	3 (0–5)	3 (0–4)	1.11 (0.75–1.67)	0.601	1.04 (0.65–1.68)	0.865	3 (0–4)	2 (0–4)	1.17 (0.65–2.08)	0.606
*Secondary outcomes*										
mRS 0–1 at 90 d	67/151 (44.4)	66/146 (45.2)	1.03 (0.66–1.63)	0.885	0.95 (0.53–1.71)	0.867	33/71 (46.5)	46/73 (49.3)	1.12 (0.58–2.16)	0.734
mRS 0–2 at 90 d	74/151 (49.0)	72/146 (49.3)	1.01 (0.64–1.60)	0.958	0.94 (0.51–1.72)	0.835	35/71 (49.3)	38/73 (52.1)	1.12 (0.58–2.15)	0.741
mRS 0–3 at 90 d	88/151 (58.3)	89/146 (61.0)	1.12 (0.70–1.78)	0.638	0.91 (0.51–1.63)	0.756	43/71 (60.6)	39/73 (53.4)	0.77 (0.39–1.45)	0.388
DCI	27/143 (18.9)	25/136 (18.4)	0.97 (0.53–1.77)	0.915	1.02 (0.41–2.54)	0.967	13/69 (18.8)	15/70 (21.4)	1.18 (0.51–2.70)	0.704
*Safety outcomes*										
Death within 90 d	20/151 (13.3)	13/146 (8.9)	0.64 (0.31–1.34)	0.237	0.65 (0.25–1.67)	0.370	10/71 (14.1)	4/73 (5.5)	0.35 (0.11–1.19)	0.092
Any ICH	34/149 (22.8)	28/143 (19.6)	0.82 (0.47–1.45)	0.499	0.97 (0.44–2.15)	0.938	11/71 (15.5)	15/73 (20.6)	1.41 (0.60–3.33)	0.432
Symptomatic ICH	8/149 (5.4)	6/142 (4.2)	0.78 (0.26–2.30)	0.649	0.86 (0.31–2.41)	0.771	3/71 (4.2)	2/72 (2.8)	0.65 (0.11–4.00)	0.640

ASPECTS, Alberta stroke program early computed tomography score; AOR, adjusted odds ratio; aP, adjusted P; CI, confidence interval; CTA, computed tomography angiography; DCI, dramatic clinical improvement; ICH, intracranial hemorrhage; IQR, interquartile range; MRI, magnetic resonance imaging; mRS, modified Rankin scale; NCCT, non-contrast computed tomography; NIHSS, National Institutes of Health Stroke Scale; OR, odds ratio.

a Adjusted for age, baseline NIHSS score, ASPECTS, last known well to arterial puncture time, occlusion site, successful reperfusion and centers.

b Defined as NIHSS score ≤ 1 at 24 h or ≥ 10 points drop within 24 h.

c According to the heidelberg bleeding classification.

All outcome measures of PS matched population were consistent with mixed-effects logistic regression models in the prematched population, which is also presented in Table 2 and Figure 2, with no significant difference in 90-day functional disability (ordinal mRS shift: OR 1.25, 95% CI [0.76–2.04], *p* value = 0.379), mRS 0-1(OR 1.07, 95% CI [0.61–1.87], *p* = 0.813), mRS 0–2 (OR 1.25, 95% CI [0.72–2.18], *p* = 0.436), mRS 0–3 (OR 1.30, 95% CI [0.73–2.32], *p* = 0.370), DCI (OR 1.38, 95% CI [0.69–2.77], *p* = 0.359), rate of ICH (21.7% vs. 25.3%, *p* = 0.552), sICH (7.2% vs. 5.1%, *p* = 0.541), and rate of mortality at 90 day (13.3% vs. 7.9%, *p* = 0.224) across the MRI and the NCCT ± CTA groups in the primary study cohort, the analysis of the Guideline-like cohort yielded similar results.

## Discussion

Analysis of patients in ANGEL-ACT registry showed that patients who suffered ischemic stroke due to large vessel occlusion of intracranial ICA or MCA-M1/M2 underwent MT in the extended window of 6–24 h, selection by NCCT ± CTA leads to no significant difference in clinical outcomes compared with patients selected by advanced imaging of MRI. The result of our study along with others (11, 20, 21) suggests that simplified imaging selection and widen criteria of MT for patients presenting in the extended window might be reasonable. Prospective randomized clinical trials are needed to confirm the results.

The role of CTP in selecting patients who may benefit from MT has been studied in several reports (10, 14, 22), the impact of using MRI to identify those population are not extensively studied. In the United States, CTP is more often used than MRI to select patients in the extended window for MT, because it is more widely available. In the DEFUSE 3 trial (9), 73% of patients underwent CTP compared with and 27%patients underwent MRI. In the Trevo Registry, 34.5% underwent CTP compared with 0.6% patients underwent MRI (14). This does not seem to be the case in China where MRI seems more prevalent. ANGEL-ACT is a prospective multicenter registry conducted at 111 hospitals between November 2017 and March 2019 in China. In the ANGEL-ACT registry, the decision of which imaging modality should be used for selecting patients is made by physicians at each sub site. MRI is more often used in the ANGEL-ACT registry for multiple reasons below: 1) Limited staff availability in the off-hours for CTP contrast injection and image processing. 2) the cost of CT/CTA/CTP is much higher compared with MRI + MRA. 3) Formal written consent is required in China for iodine contrast injection, which is necessary for CTA/CTP but not for MRI. This circumstance provides an opportunity for us to compare the MRI based patient selection and NCCT ± CTA based selection.

There are several advantages of MRI over CTP. Compared with using relative CBF of CTP map to identify ischemic core, MRI seems more accurate, for variability between CBF core volumes and irreversibly injured tissue can occur for a variety of reasons (23). Ischemic core estimates of relative CBF are based on severe reductions in blood flow, when collateral circulation from leptomeningeal anastomosis or capillary starts feeding irreversibly injured tissue, which is more prevalent in the extended window, CTP mapping with relative CBF <30% may not be able to identify irreversible tissue where reperfusion occurred. This could lead to underestimation of the ischemic core. In both SWIFT PRIME and DEFUSE 3 trials, MRI-selected patients had a slightly higher rate of favorable outcomes than CTP-selected patients. Whereas, lesions on DWI or ADC sequence directly reflect the state of the brain tissue and are used as golden standard in CTP studies to decide an optimal threshold for identifying ischemic core (24). Moreover, analysis of the impact of different imaging modalities found there were no significant difference in hospital arrival to femoral puncture times in DAWN, DEFUSE 3 and SWIFT PRIME trials (1, 8, 9).

The aim of additional CTP or MRI criteria in DAWN and DEFUSE 3 is to identify patients with smaller ischemic core, patients with smaller ischemic core and larger ischemic penumbra have more chance to obtain good outcomes. As MT is becoming widely adopted, concerns on the rationality of the extra imaging criteria raised. Studies on the effect of CTP for patients underwent MT in the extended time window suggest CTP may not improve patients’ outcome (11, 20, 21). After balancing baseline characteristics by using PS matching, our study showed no significant difference in any clinical outcome between patients selected by NCCT ± CTA and patients selected by MRI as well. These studies, combined with ours, implicitly suggest that the criteria in the DAWN and DEFUSE 3 might be so stringent that it might exclude patients who can potentially benefit from this therapeutic strategy. The reason underlying such a result worth more exploratory research. On one hand, there are studies suggesting that even patients with large ischemic core can also benefit from MT (10), current ongoing trials (25, 26) investigating the efficiency of MT for large core stroke patients will further clarify this issue, on the other, the histological changes of ischemic stroke in the extended window needs to be further clarified. Moreover, LKW-to-arterial puncture time, which is a crucial workflow metric of stroke care, is significantly shorter in patients selected by NCCT ± CTA alone compared with patients selected by MRI, previous studies on CTP showed similar results (27). This suggests that a pragmatic criterion for selecting patients presenting in the extended window with NCCT ± CTA to receive MT may decrease delays in the stroke care workflow without diminishing the chance of obtaining a good outcome nor increasing risks of post-surgical complications like ICH. This is also meaningful for promoting MT to where advanced imaging modalities are not available.

Our findings should be interpreted based on the following limitations. First, this is a retrospective analysis of a prospective registry, imaging modality (CTP or MRI) that is used to select patients for MT was determined by the physicians at each site, there are no explicit criteria of which imaging modality should be used to select patients. Second, the ANGEL-ACT registry is a real-world multicenter registry that eliminated the potential selection bias imposed by the inclusion criteria in prospective clinical trials. The baseline characteristics are not evenly distributed among the groups, propensity-matched analysis is performed to balance confounding factors between groups in both cohorts to improve comparability, a large number of patients were excluded though. Third, the MRI protocols were not standardized before recruiting subjects, thus varying by sites and equipment manufacturers, which may lead to lack of uniformity on image collection and ischemic core volume assessments. Fourth, there are no data on patients who received medical management alone in the ANGEL-ACT registry, randomized controlled studies comparing two therapy arms are necessary. Fifth, the universality of the conclusion drawn by our analysis is uncertain, which means whether the practice of skipping ischemic core quantification could be universally adopted needs to be further verified.

## Conclusion

Our study showed that in patients who suffered ischemic stroke due to large vessel occusion of Intracranial ICA or MCA-M1/M2 underwent MT in the extended window of 6–24 h, selection by NCCT ± CTA leads no significant difference in clinical and radiographic outcomes compared with patients selected by advanced imaging modality of MRI. Simplified imaging selection and widen criteria of MT for patients presenting in the extended window might be reasonable. Prospective randomized clinical trials are needed to confirm the results.

## Data availability statement

The raw data supporting the conclusions of this article will be made available by the authors, without undue reservation.

## Ethics statement

Written informed consent was obtained from the individual(s) for the publication of any potentially identifiable images or data included in this article.

## Author contributions

ZM designed, led the ANGEL-ACT registry and had full access to all of the data in the study and took responsibility for the integrity of the data and the accuracy of the data analysis. HC and ZY prepared the first draft of the report. All authors contributed to the article and approved the submitted version.

## Funding

This study is supported by National Key Research and Development Program of China (2016 YFC1301500), The clinical significance of balloon catheter in the process of clipping complex intracranial aneurysms (LHGJ20210874) and Significance of balloon catheter in intracranial aneurysm clipping (2022C01SF023).

## Conflict of interest

The authors declare that the research was conducted in the absence of any commercial or financial relationships that could be construed as a potential conflict of interest.

## Publisher’s note

All claims expressed in this article are solely those of the authors and do not necessarily represent those of their affiliated organizations, or those of the publisher, the editors and the reviewers. Any product that may be evaluated in this article, or claim that may be made by its manufacturer, is not guaranteed or endorsed by the publisher.
