# Supplementary material for: Does MRI add value in selecting patients for thrombectomy beyond the 6 h window? A matched-control analysis
Source: Front Neurol. 2023 Apr 17;14:1135624. doi: 10.3389/fneur.2023.1135624 (PMC10149695; doi:10.3389/fneur.2023.1135624)
Supplement: Supplementary file 1 [file Table_1.docx]

**Supplementary table 1. Comparison of matched and unmatched Extended Time Window Patients**

| **Primary Study Cohort** | **PS matched population**  **(n=204)** | **PS unmatched population**  **(n=220)** | **P value** |
| --- | --- | --- | --- |
| Age, median (IQR), years | 65 (55-71) | 63 (52-70) | 0.361 |
| Male sex | 139/204 (68.1) | 152/220 (69.1) | 0.835 |
| Baseline mRS score |  |  | 0.149 |
| 0 | 181/204 (88.7) | 190/220 (86.4) |  |
| 1 | 23/204 (11.3) | 26/220 (11.8) |  |
| 2 | 0/204 (0) | 4/220 (1.8) |  |
| Baseline NIHSS score, median (IQR) | 15 (12-19) | 14 (9-19) | 0.464 |
| NCCT/DWI ASPECTS, median (IQR) | 8 (7-10) | 8 (6-10) | 0.962 |
| Volume, median (IQR), ml | - | - | - |
| Occlusion site |  |  | 0.905 |
| Internal carotid artery | 58/204 (28.4) | 65/220 (29.6) |  |
| MCA-M1 | 127/204 (62.3) | 137/220 (62.3) |  |
| MCA-M2 | 19/204 (9.3) | 18/220 (8,2) |  |
| Intravenous thrombolysis | 44/204 (21.6) | 54/220 (24.6) | 0.491 |
| General anesthesia | 125/204 (61.3) | 130 (59.1) | 0.692 |
| Successful reperfusion* | 185/204 (90.7) | 188/220 (85.5) | 0.103 |
| Pass number of thrombectomy, median (IQR) | 1 (1-2) | 2 (1-3) | 0.598 |
| LKW-to-arterial puncture time, median (IQR), min | 525 (415-723) | 475 (408-658) | 0.159 |
| Puncture-to-reperfusion time, median (IQR), min | 85 (54-121) | 90 (50-134) | 0.456 |
| **Guideline-Like Cohort** | **PS matched population**  **(n=146)** | **PS unmatched population**  **(n=157)** | **P value** |
| Age, median (IQR), years | 64 (52-71) | 63 (53-70) | 0.863 |
| Male sex | 101/146 (69.2) | 105/157(66.9) | 0.712 |
| Baseline mRS score |  |  | 0.466 |
| 0 | 128/146 (87.7) | 142/157 (90.5) |  |
| 1 | 18/146 (12.3) | 15/157 (9.6) |  |
| Baseline NIHSS score, median (IQR) | 15 (12-19) | 15 (11-19) | 0.652 |
| NCCT/DWI ASPECTS, median (IQR) | 9 (7-10) | 9 (7-10) | 0.002 |
| Volume, median (IQR), ml | - | - |  |
| Occlusion site |  |  | 1.000 |
| Internal carotid artery | 44/146 (30.1) | 48/157 (30.6) |  |
| MCA-M1 | 102/146 (69.9) | 109/157 (69.4) |  |
| Intravenous thrombolysis | 27/146 (18.5) | 40/157 (25.5) | 0.167 |
| General anesthesia | 93/146 (63.7) | 93/157 (59.2) | 0.479 |
| Successful reperfusion* | 130/146 (89.0) | 139/157 (88.5) | 1.000 |
| Pass number of thrombectomy, median (IQR) | 2 (1-3) | 1 (1-2) | 0.674 |
| LKW-to-arterial puncture time, median (IQR), min | 499 (419-695) | 470 (405-663) | 0.198 |
| Puncture-to-reperfusion time, median (IQR), min | 88 (53-130) | 80 (51-123) | 0.685 |

Abbreviations: ASPECTS, Alberta Stroke Program Early Computed Tomography Score; CTA, computed tomography angiography; DWI, diffusion-weighted imaging; IQR, interquartile range; LKW, last known well; M1, M1 segment; M2, M2 segment; MCA, middle cerebral artery; MRI, magnetic resonance imaging; mRS, modified Rankin scale; NCCT, non-contrast computed tomography; NIHSS, National Institutes of Health Stroke Scale; PS, propensity score.

* Successful reperfusion was defined as a modified Thrombolysis in Cerebral Infarction score of 2b or 3.
